# Editorial: Acidophile microbiology: from extreme environments to biotechnological applications

**DOI:** 10.3389/fmicb.2024.1454559

**Published:** 2024-07-04

**Authors:** Elizabeth L. J. Watkin, Ivan Nancucheo, Axel Schippers

**Affiliations:** ^1^School of Science, Edith Cowan University, Joondalup, WA, Australia; ^2^Faculty of Engineering, Architecture and Design, Universidad San Sebastián, Concepción, Chile; ^3^Department of Geomicrobiology, Federal Institute for Geosciences and Natural Resources, Hannover, Germany

**Keywords:** acidophile, extremophile, bioleaching, bioremediation, microbial diversity

Acidophiles are found in a surprisingly wide range of environments that would be inhospitable to most life forms and they possess a wide range of adaptions to these extreme conditions. While their resilience and metabolic versatility in such hostile conditions offer invaluable insights into evolutionary adaptation and biochemical diversity, they also provide valuable enzymes and metabolic pathways that can be harnessed for various industrial purposes. This Research Topic covers 18 manuscripts that provide a comprehensive overview of acidophile microbiology, from basic research to biotechnology, and highlights the potential of acidophilic microorganisms as valuable resources for environmental and industrial applications.

From a practical standpoint, acidophile microbiology is pivotal in several industrial sectors, most notably in biomining. Acidophiles play a critical role in biomining processes by catalyzing the oxidative dissolution of metal sulfides as well as the reductive dissolution of laterites, thus facilitating the extraction of valuable metals from ores while minimizing the use of chemical leaching agents compared to traditional ore processing methods. A number of manuscripts examined acidophiles in the context of bioleaching and mineral dissolution.

The influence of microbial community and solution redox potential on the bioleaching of a sulfide mineral concentrate rich in the less studied mineral tennatite was investigated using activated carbon (Kondo et al.). Another study addressed the question if the nutrient content in the often used microbial growth media is appropriate for optimal bioleaching and identified overdosing as a problem (Falagan et al.). A challenge in biomining is to cope with high sulfate concentrations and a biochemical study of *Leptospirillum ferriphilum* investigated its osmotic response (Arias et al.).

Relevant for reductive bioleaching of limonitic laterites, the ferric iron bioreduction kinetics was studied in one case (Hubau et al.). while the dissolution of the most abundant mineral goethite in these ores was studied with different acidophiles (Stankovic and Schippers). A further study described that there is a negligible impact of contaminating Fe(II)-oxidizing *Leptospirillum* sp. on the efficiency of aerobic laterite bioleaching with *Acidithiobacillus* sp. (Hetz and Schippers).

While bioleaching is traditionally associated with autotrophic acidophiles a number of heterotrophic ones have also been shown to reduce Fe(III). Gonzalez et al. reviewed the possible application of acidophilic heterotrophs in situations where oxidative bioleaching is ineffective.

Biofilm formation is an important first step in the attachment of microorganisms to the mineral surface to facilitate mineral leaching. Rossoni et al. demonstrated the importance of membrane vesicles in the attachment behavior of the extreme acidophile, “*Fervidacidithiobacillus caldus.”*

The metabolic pathways of acidophiles can also be utilized with secondary resources of metals such as lithium-ion batteries which contain substantial amounts of critical minerals. Lalropuia et al. used a two-step bioleaching process utilizing microorganisms enriched from a metal contaminated pit lake.

Acidophilic microorganisms also have application in the bioremediation of acid-contaminated environments. Their ability to metabolize toxic heavy metals and degrade organic pollutants in acidic wastewaters underscores their potential in eco-friendly remediation strategies. However, caution must be exercised with Rosendahl et al. querying the sustainability of *in-situ* bioremediation of uranium contaminated sites after demonstrating the mobilization of U by *At. ferrooxidans* and effects on U isotope fractionation.

Acidophilic conditions result in unique evolutionary pressures leading to the development of novel enzymes and proteins. These acidophile-derived enzymes exhibit unparalleled stability and activity under acidic conditions and can find utility in diverse industrial processes. A review of the unique enzyme Tetrathionate hydrolase by Kanao describes the enzyme that is found in acidophilic sulfur oxidizing microorganisms and catalyzes the hydrolysis of tetrathionate. The structural properties and regulatory mechanisms are discussed. The novelty of enzymes identified in acidophiles also result in adaptations compared to their neutrophilic counterparts. Through the characterization of three recombinant periplasmic redox proteins from the moderate acidophile *Ferrovum* sp. PN-J47-F6 Ullrich et al. showed a gradual adaption of redox proteins, becoming more positive with decreasing pH. A pangenome-level analysis of nucleoid-associated proteins in the Acidithiobacillia class gave insights into their functional roles in mobile genetic elements biology (Beard et al.).

Beyond industrial applications, acidophile microbiology contributes significantly to our understanding of microbial diversity and adaptation strategies in extreme environments. The genomic and metabolic diversity among acidophiles not only broadens our taxonomic knowledge but also provides insights into universal principles governing microbial survival and evolution in challenging habitats. A number of diverse acidic environments ranging from high to low temperature and saline ones were the study of several papers. Boase et al. investigated the microbial diversity of acidic saline lakes found in Western Australia identifying multiple organisms with potential in biomining. Yellowstone National Park was the focus of a study by Kim et al. who isolated and characterized two strains of *Alicyclobacillus* able to tolerate both extreme temperature and low pH. These species were demonstrated to have unique metabolic adaptions to these extreme conditions. The novel genus “*Igneacidithiobacillus*” is described by Arisan et al. from genetic and genomic diversity and distribution patterns of both characterized and uncharacterized sequence data obtained from the geothermally active sites across the Pacific ring of fire. On the opposite climatic extreme, psychrotolerant strains of *Acidithiobacillus ferrooxidans* from polar and subpolar environments were characterized by Muñoz-Villagrán et al.. Cuevas et al. described the microbial communities and resultant nutrient cycling in an acidic glacial lake in Patagonia demonstrating active communities that contributed significantly to nutrient utilization and mobilization. These studies explore the unique environments with multiple stresses and the novel microorganisms that exist there. They also demonstrate the existence of acidophilic isolates that can tolerate multiple stresses to cope with the multiplicity of environments in which bioleaching may occur.

Acidophile microbiology exemplifies how extreme environments foster biological innovation and biotechnological advancement. By deciphering the intricate adaptations of acidophiles and translating them into practical applications, not only the frontiers of microbiology are expanded but also a more sustainable and technologically advanced future is possible.

## Author contributions

EW: Writing – original draft, Writing – review & editing. IN: Writing – review & editing. AS: Writing – review & editing.

